# Letter from Budapest, Hungary

**Published:** 1890-03

**Authors:** Otto Marx

**Affiliations:** Budapest, Hungary


BUDAPEST, HUNGARY, January 31st, 1890.

EDITOR DENTAL REGISTER.

*Dear Sir*:—Upon your request I promised to send you something for the readers of the REGISTER pertaining to the state of dentistry in Hungary. At the time I did not fully realize what I promised, and the fulfillment of the promise has been delayed longer than I intended, but I will now endeavor to comply with my obligations in this respect.

The history of dentistry in this country hardly dates back a half century, and in order to comprehend how weak and how slow the progress has been since then, it is necessary first to give you a brief sketch of the universities in this country.

The total population at that time amounted to 16,000,000 inhabitants and there were just two universities in the country. The one university up to 1858 only educated and gave diplomas for "surgeons" or doctors of second rank, or in other words, educated "barbers" for military service. The other university, the one in Budapest, had two sorts of diplomas—one for the barber, as mentioned above, and one for the higher class of students who received the degree of M.D. All these, however, had to be graduates of some good school or college. This, however, has all been vastly improved since the time of Marie Theresa. There has never been, nor does there exist now, a school for dentistry in this country, neither does there exist one in Austria, *i. e.*, in Vienna, despite the splendid university there.

It has been the intention for some time of the university to establish a department of dentistry here in Pest. But as the parliament has only appropriated 1,000 florins a year (less than $500) with which to pay the one professor, assistants, and servants, besides buying the necessary outfit and other paraphernalia indispensable to a school of the most unpretending kind.

From the above it will be seen that so far very little has been done for the promotion of dentistry here. Dentistry in Hungary really takes its origin from two military surgeons who came here from Austria after the revolution of '48. They obtained their knowledge in the office of some dentists in Vienna. The whole knowledge of dentistry here at that time consisted in extracting and substituting the natural teeth with artificial ones on metallic bases. Filling teeth was very little known and termed by those who had heard of it as a humbug. Pest had at this time a population of 200,000 and not only these, but the whole of Hungary, depended upon these two surgeons and one barber for their dental operations.

The business of the dentist at this time was to make artificial dentures, the barber performed all the operations, and even to this day you can have teeth extracted in any barber shop. From this it will be easily seen why "dentistry" fell into the hands of the jewellers; they were skilled mechanics.

In 1856 the university appointed one man, an M.D., (who, by the way, had never studied dentistry abroad and of course not at home as there were no schools) as professor of dentistry. This gentleman up to 1883 gave courses in dentistry. These courses consisted of lectures once a week for six months; he gave these lectures *not* in his office but at his home. He lectured chiefly on the anatomy of the teeth and occasionally he made a plaster model. Generally his class was composed of half a dozen medical students. If any of these wished to see how a tooth was soldered to the plate, or any other work performed, he had secretly to bribe the "technicher" and so get the desired permission. So matters stood even as late as the last four or five years.

The gentleman from whom I have this information was the first to go abroad, namely, to London, and there spend a year in the dental college. This was in 1861. As the years rolled by the number of dentists in Hungary increased from three to three hundred. Of these hardly a third are doctors or graduated dentists. The most remarkable thing is the way in which the diplomas for dentistry were given out here. Any doctor or surgeon could obtain a degree of "Mogisten der zahnheilkunde" by merely passing a special examination in anatomy, in which examination, however, no mention was made of "zahnheilkunde." The state of affairs in this respect, I am sorry to say, has not improved for even every physician is at liberty to practice dentistry if he so desires without any further hindrance. In case of a foreigner or a doctor who has received his diploma (be it M. D., or D.D.S., in another country) it is different, he has to have his diploma recognized and to pass an examination on the anatomy of the head and surgery of the mouth; of the "zahnheilkunde," proper, however, no questions are asked, because the examiner (the dean of the college) hasn't the faintest idea of dentistry.

Budapest, the capital of Hungary, has a population of half a million, with a splendid university in which there are from 800 to 900 medical students, but the chair of dentistry is filled by one man who in his private clinic instructs sixty or seventy medical students once or twice a week on care of the teeth and occasionally gives them an opportunity to see a tooth extracted. The filling of teeth, however, is not taught at all; to get even the slightest knowledge of this they have to pay some practicing dentist to be allowed to see and pick up what they can around the office. The gentleman who gave me this information was the first to import a dental engine into this country, also the first to insert a gold filling, and this in 1865.

The standing of the profession is, however, thanks to foreign influence (in the way of journals, dental dealers, and American and English dentists), improving; the school for dentistry here, however, is made remarkably conspicuous by its absence.

Very truly yours, OTTO MARX.

